# Posterior Tibial Slope Characteristics and Their Relationships With Thigh Muscle Morphology, Activation Patterns, and Dynamic Knee Valgus in Male and Female Soccer Players Before and After Fatigue: A Descriptive Laboratory Study

**DOI:** 10.1177/23259671251350303

**Published:** 2025-07-01

**Authors:** Gerda Strutzenberger, Florian B. Imhoff, Jakob Ackermann, Laura Zehnder, Daniel P. Fitze, Sandro F. Fucentese, Johannes Scherr, Stefan Fröhlich, Jörg Spörri

**Affiliations:** * Sports Medical Research Group, Department of Orthopedics, Balgrist University Hospital, University of Zurich, Zurich, Switzerland; † Motion Analysis Zurich, Department of Orthopedics, Balgrist University Hospital, Children’s Hospital, University of Zurich, Zurich, Switzerland; ‡ University Centre for Prevention and Sports Medicine, Department of Orthopedics, Balgrist University Hospital, University of Zurich, Zurich, Switzerland; § Department of Medical, Health & Sports Engineering, Management Center Innsbruck, Innsbruck, Austria; ‖ Knee Surgery, Department of Orthopedics, Balgrist University Hospital, University of Zurich, Zurich, Switzerland; Investigation performed at the Department of Orthopedics, Balgrist University Hospital, Zurich, Switzerland

**Keywords:** athlete, football, knee injuries, primary prevention, surgery

## Abstract

**Background:**

Soccer players are at high risk of anterior cruciate ligament injury as well as reinjury, with higher rates in women and in the supporting leg, and fatigue impairs the neuromuscular control of the knee joint. However, little is known about the bony anatomy of the knee joint and its relationship with neuromuscular measures related to knee stabilization, and studies investigating anatomic and biomechanical measures simultaneously are widely lacking.

**Purpose:**

To investigate (1) whether posterior tibial slope (PTS) characteristics and thigh muscle morphology differ between sexes; (2) whether thigh muscle activation patterns and dynamic knee valgus differ between sexes and fatiguing states during a drop jump (DJ); and (3) whether certain relationships among PTS characteristics, thigh muscle morphology, thigh muscle activation patterns, and dynamic knee valgus exist.

**Study Design:**

Descriptive laboratory study

**Methods:**

Magnetic resonance images of 32 healthy national-level soccer players (15 female, 17 male) were acquired, and their PTS characteristics and thigh muscle anatomic cross-sectional areas (CSAs) were determined. Muscle activation patterns, as well as dynamic knee valgus (quantified as medial displacement of the knee joint center [medial knee displacement, or MKD] in the anatomic frontal plane), were assessed via surface electromyography and 3-dimensional motion capture while participants performed DJs before and after a fatigue protocol.

**Results:**

Female players had smaller hamstring and quadriceps CSAs (*P* < .001). During DJ landing, female players demonstrated significantly greater hamstring activity (*P* = .04). There were significant sex differences in the lateromedial hamstring activation ratio (*P* = .03), with female players exhibiting greater medial than lateral hamstring activation (ratio <1.0), whereas male players presented opposite activation patterns (ratio >1.0). MKD was greater in female players than in male players (*P* = .02). Moreover, in female players, the steeper their lateral PTS was, (1) the greater their quadriceps-to-hamstring activation ratio and the lower their hamstring activity, (2) the smaller their lateromedial hamstring activation ratio, and (3) the lower their MKD. In male players, a steeper lateral PTS was associated with greater quadriceps activity only. No fatigue-induced differences were observed.

**Conclusion:**

Among soccer players, thigh muscle morphology, muscle activation patterns, and dynamic knee valgus during DJ landing are sex dependent. Moreover, there are distinct sex-specific associations between PTS characteristics, thigh muscle morphology, and measures related to neuromuscular control of the knee joint.

**Clinical Relevance:**

These study results may further highlight the importance of also considering sex and the bony anatomy of the knee joint in the design of neuromuscular prevention and rehabilitation, or the choice of graft type. However, future studies are needed to assess the effects of sex- and/or knee anatomy–dependent prevention efforts or graft choice on clinical outcomes.

Pivoting-sports athletes, such as soccer players, are at relatively high risk of anterior cruciate ligament (ACL) injury and reinjury,^6^ with higher rates in female players, and with female players’ being more likely to injure the ACL in their supporting leg.^6,8,62^ In addition to sex and leg dominance, fatigue may also play a crucial role in ACL injury, as it increases dynamic knee valgus (ie, combinatory motion of hip adduction and internal rotation, knee abduction, tibial external rotation and anterior translation, and ankle eversion^25^) and fosters stiff landing, increasing tibial anterior shear forces.^60^ In this context, it has been reported that match-induced eccentric hamstring muscle fatigue is greater in females than in males,^45^ and fatigue particularly impairs neuromuscular knee control in the nondominant (typically supporting) leg.^2,27^ Moreover, available research suggests that altered movement patterns that place the ACL at risk may best be identified in a fatigued state but, under certain circumstances, may already be identified in a standard dynamic assessment.^44^

Recent studies have identified a specific characteristic of the bony anatomy of the knee joint—that is, a steeper posterior tibial slope (PTS)—as a potential risk factor for ACL injuries and reconstruction failure.^15,23,26,61^ Biomechanical studies have revealed that anterior tibial translation and internal rotation of the tibia are both influenced by the extent of the PTS.^5,32,55^

Noncontact ACL injuries commonly occur during jump landing or cutting impacts, in which the quadriceps muscles are suddenly and aggressively activated^14^ and the knee is forced into biomechanically unfavorable positions where the ACL is maximally stretched, such as dynamic knee valgus and internal or external rotation of the tibia.^9,33,46^ In such cases, a steeper lateral PTS may further increase aggressive quadriceps activation and anterior tibial translation, as well as (when combined with knee valgus) internal tibial rotation.^5^ In turn, adequate quadriceps and hamstring co-contraction and adequate dynamic knee valgus control may counteract the aforementioned ACL-threatening loading patterns.^36,44^

Accordingly, the bony anatomy of the knee joint,^10^ the morphology of the thigh muscles,^1,39^ and measures related to knee joint stability in the sagittal plane (eg, thigh muscle activation patterns^17,52^) and stability in the frontal plane (eg, dynamic knee valgus^13,18,22,31,48^) have been extensively studied, especially with respect to sex-specific and fatigue-related differences. However, little is known about sex differences in the bony anatomy of the knee joint and thigh muscle morphology. Despite some evidence with respect to neuromuscular control strategies for the knee joint in dependency of sex and fatigue, studies investigating anatomic and biomechanical measures simultaneously are widely lacking.

Given a specific knee anatomy, the neuromuscular system that is responsible for knee stability is likely to cope with unfavorable knee joint anatomies and to better stabilize the knee joint. Such coping may manifest in both structure (ie, thigh muscle morphology) and function (ie, muscle activation patterns), both of which are known to influence muscular strength.^54^ In this context, it has already been shown that healthy individuals with a greater lateral PTS have a muscle morphology profile that includes a larger lateral (ie, biceps femoris long head) volume,^51^ which is usually assessed by the proxy measure of the anatomic cross-sectional area (CSA). However, whether and how the activation patterns of the entire thigh musculature (ie, the quadriceps and hamstring muscles) cope with such knee anatomy has not yet been elucidated, nor whether this also affects the associations with dynamic knee valgus.

Therefore, the purposes of this study were to investigate (1) whether the bony anatomy of the knee joint (ie, the PTS characteristics and the thigh muscle morphology) differ between sexes; (2) whether the thigh muscle activation patterns and the amount of dynamic knee valgus differ between sexes and fatiguing states during a drop jump (DJ); and (3) whether certain relationships between PTS characteristics, thigh muscle morphology, thigh muscle activation patterns, and dynamic knee valgus exist. Ultimately, such knowledge may have potential implications for the design of neuromuscular prevention programs and/or ACL graft choices.

## Methods

### Study Design and Participants

In this descriptive laboratory study, 15 female and 17 male soccer players (female: Swiss National League A and B; male: Swiss Football League) aged ≥16 years were included. According to the classification system of McKay et al,^40^ this corresponds to “highly trained/national-level athletes.” The soccer players were recruited during the winter season breaks through official enquiries to their soccer clubs, which referred interested players to us. Exclusion criteria for study participation included previous ACL tear in any knee and any previous knee, ankle, or hip surgery. Participants were excluded if functional scores indicated any impairment (Tegner < 9, International Knee Documentaton Committee < 100%, Lysholm < 100).^29,35,56^ After performing the fatigue protocol described below, the data from 2 female players (both limbs) and 3 male players (nondominant limbs) had to be removed because their sweating resulted in inadequate electromyography (EMG) signals (eg, slipping of the electrode). Thus, for analyses including only prefatigued data, the entire cohort was used; for analyses including fatigued data, the reduced subset was used. A detailed overview of the characteristics of the subsamples on which the analysis of this study is based is presented in Table 1. All but 2 soccer players reported that their right leg was their dominant leg, defined as their kicking leg.

**Table 1:** Overview of Characteristics of Subsample of Participants Analyzed in the Prefatigued and Fatigued States*^*a*^*

		Age, y	Height, cm	Weight, kg	Participants With Available Data for Dominant Leg, n	Participants With Available Data for Nondominant Leg, n
Female (n = 15)	Prefatigued	21.4 ± 3.0	167.0 ± 7.1	62.9 ± 9.6	15	15
	Fatigued	21.9 ± 3.3	165.8 ± 7.1	61.7 ± 7.4	13	13
Male (n = 17)	Prefatigued	22.3 ± 2.9	178.6 ± 6.8	74.2 ± 8.3	17	16
	Fatigued	22.3 ± 2.9	178.6 ± 6.8	74.2 ± 8.3	17	13

a Data are presented as mean ± SD unless otherwise indicated.

The underlying study protocol was approved by the institutional review board at Balgrist University Hospital and the cantonal ethics committee Zurich (KEK-ZH-NR: 2020-02583). The participants approved and signed the consent form before participation. All procedures were in accordance with the Declaration of Helsinki and national laws.

### Radiological Assessment

Each study participant underwent magnetic resonance imaging (MRI) with a standardized protocol (3-Tesla; MAGNETOM Prisma; Siemens Healthcare). Both knees were imaged using a musculoskeletal protocol that included the following sequences—dimensionality, orientation and contrast weighting (repetition time and echo time, ms): 2-dimensional (2D) coronal proton density fat saturation (PD-FS) (5000-5300; 36.0); 2D coronal T1-weighted (620-670; 8.9); 2D sagittal PD-FS (5500-6000; 36.0); 3-dimensional (3D) axial water excitation Double Echo Steady State (11.0; 3.8; slice thickness, 1.0 mm; flip angle, 28°); slice thickness and interslice gap of the 2D sequences: 3.0 and 0.6 mm.

**Figure 1.**
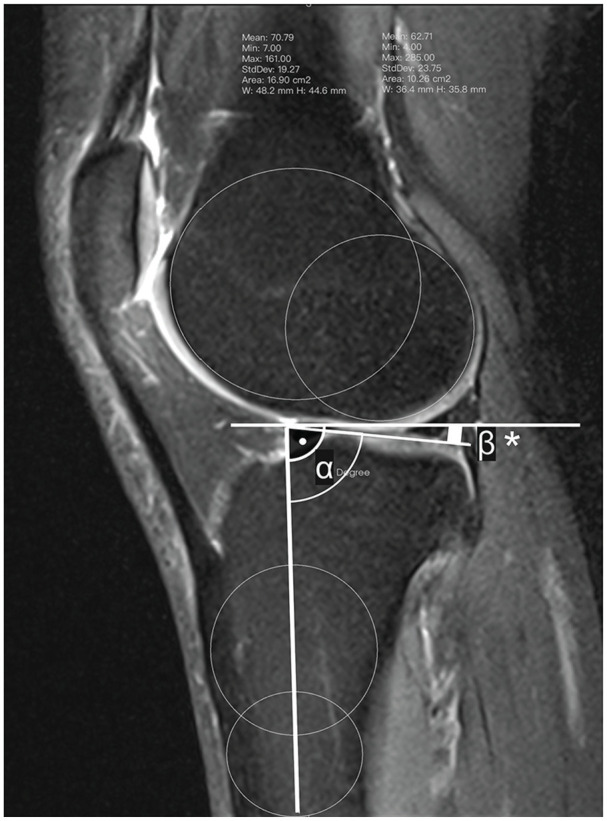
Example of lateral posterior tibial slope measurements on a sagittal view of the lateral compartment of the knee: slope angle β* (°) = 90°–α (°). The solid dot in the figure refers to a right angle (i.e., 90°).

Anatomic parameters of the bony anatomy of the knee joint, such as the medial PTS (MPTS) and the lateral PTS (LPTS) angles, were measured in 3 steps as described in more detail elsewhere.^28^ First, the central sagittal image was determined as the image in which the tibial attachment of the posterior cruciate ligament, the intercondylar eminence, and the anterior and posterior tibial cortices appeared concave in shape. Second, 1 cranial and 1 caudal circle were positioned in the tibial head. The longitudinal axis was defined by a line that connected the centers of these 2 circles. Third, in the MRI showing the mediolateral center of the medial plateau, a tangent connecting the uppermost superoanterior and posterior cortex edges was drawn to the medial plateau, and MPTS was determined as the angle between the orthogonal to the MRI–longitudinal axis and the tangent to the medial plateau. The LPTS was determined analogously to MPTS but was based on the mediolateral center of the lateral plateau and a tangent to the uppermost even part between the superoanterior and posterior cortices. An exemplary illustration of imaging-based PTS measurements is given in Figure 1.

Each MPTS and LPTS measurement was conducted by 3 readers (1 musculoskeletal radiologist, 1 orthopaedic resident (J.A.), and 1 medical student). The interrater agreement was, according to the classification thresholds of Koo and Li,^34^ poor to moderate for single-rater values (MPTS: intraclass correlation coefficient [ICC(3,3)]; 95% CI, 0.456 [0.305-0.599]; LPTS: ICC[3,3]; 95% CI, 0.487 [0.348-0.617]) and moderate to good for the mean of 3 raters (MPTS: ICC[3,3]; 95% CI, 0.715 [0.569-0.818]; LPTS: ICC[3,3]; 95% CI, 0.740 [0.616-0.828]). For the main statistical analysis described below, the mean MPTS and LPTS values determined by the 3 raters were used.

**Figure 2.**
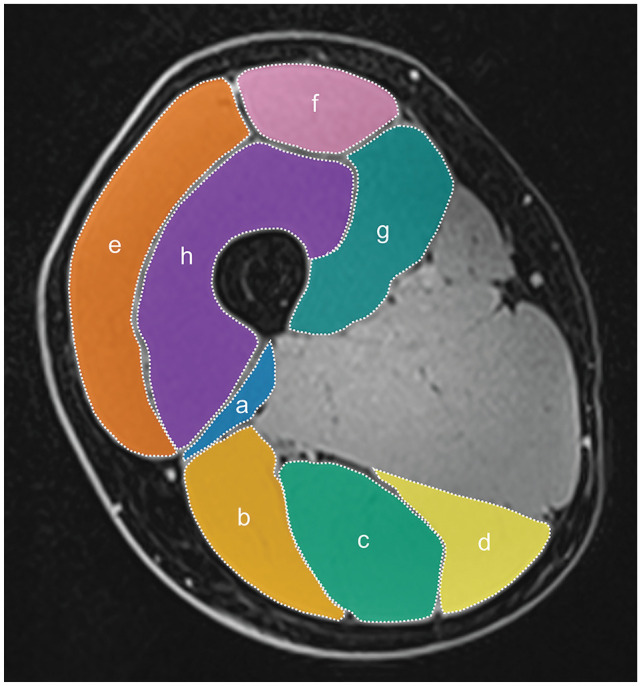
Representative image of cross-sectional area measurements on the participant’s right leg. Biceps femoris short head (*a*), biceps femoris long head (*b*), semitendinosus (*c*). semimembranosus (*d*), vastus lateralis (*e*), rectus femoris (*f*), vastus medialis (*g*), vastus intermedius (*h*).

CSA values were determined via manual segmentation of the anatomic CSAs of the hamstring (CSA_ham_) and quadriceps (CSA_quad_) muscles based on individual axial MRIs at 50% of the femoral length (which was determined on the basis of the coronal view of the lower extremities). A representative illustration of the CSA measurements is provided in Figure 2. Images were analyzed by tracing the contours of each hamstring muscle (ie, the biceps femoris short head, biceps femoris long head, semitendinosus, and semimembranosus) and quadriceps muscle (ie, the vastus lateralis, rectus femoris, vastus medialis, and vastus intermedius) using the Image J software (National Institutes of Health). These procedures have been shown to be sufficiently reliable when using panoramic ultrasound, whereas when using MRI, they can even be considered the in vivo gold standard.^49^

### Biomechanical Assessment

#### Functional Movement Task

**Figure 3.**
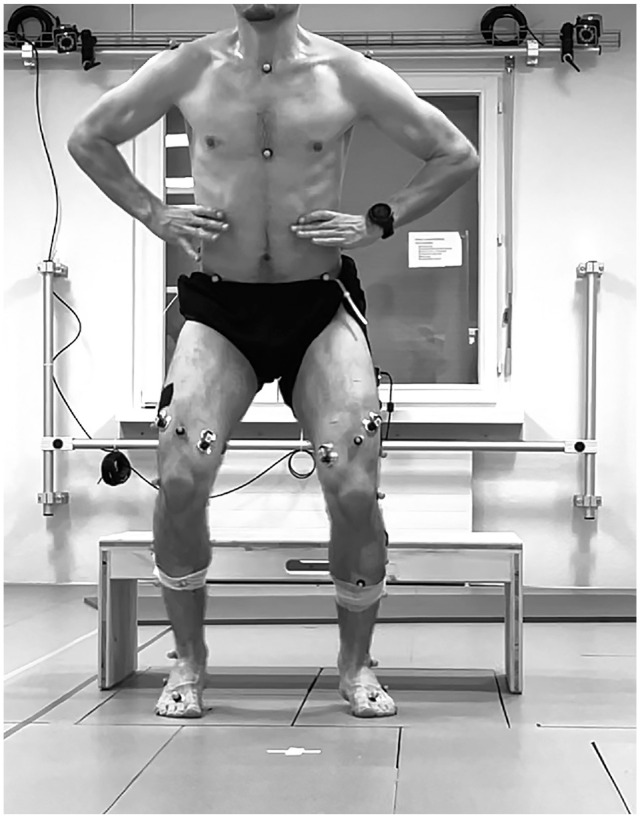
Participant performing a drop jump at the time of the first ground contact after the drop.

The participants performed 5 double-leg vertical DJs before and after the fatigue protocol, as further described below. The DJs were performed barefoot. Before data collection, familiarization trials were performed until the participants were confident. For the DJs, the participants were instructed to hold their arms akimbo, drop off a 32.6-cm box and jump off the ground as high as possible with minimal ground contact time^17^ (Figure 3).

#### Fatigue Protocol

The protocol used for the fatigue intervention was slightly adapted from that of a previous study^20^ and included both muscular and cardiovascular fatigue. One cycle included 60 seconds of jumping jacks, 30 seconds of squats with a 180° jump turn, 60 seconds of spiderman planks, 30 seconds of alternating lunge jumps, 60 seconds of alternating side lunges, and 30 seconds of sumo squats with leg curls. The tasks were performed simultaneously with an instructional video and followed each other immediately without a break. After 1 cycle, there was a 15-second break. For warm-up, participants performed 1 cycle of the fatigue protocol (described above) with submaximal intensity. As a fatiguing intervention following the functional assessments at rest, each participant performed several cycles of the fatigue protocol. The participants were asked to rate their subjective perceived fatigue level after each cycle using a visual analog scale (0: not at all fatigued, 10: extremely fatigued). The fatigue protocol was continued until one of the following occurred: the rating of subjectively perceived fatigue for the cycle was (1) scored 8 twice in a row or (2) scored 10 once. To verify the effectiveness of the fatigue protocol, the DJ height was determined immediately before and after the protocol via kinetic data collected with two 3D force plates at 2000 Hz (40 × 60 cm; Kistler 9260AA6; Kistler Holding AG) and was calculated on the basis of the vertical force signal from the initial ground contact to toe-off. Moreover, post hoc, sufficient level of fatigue was further verified by blood lactate values, which were measured immediately after the fatigue protocol (Lactate Scout 4; EKF Diagnostics; SensLab GmbH).

#### Data Collection and Postprocessing

The 3D motion patterns of the participants performing DJs were measured using a marker-based, optoelectronic 3D motion capture system with 14 infrared cameras at 200 Hz (Vero Version 2.2; Vicon; Oxford Metrics) to synchronously collect kinematic, kinetic, and surface EMG data. The process of kinematic and kinetic data acquisition and analysis was the same as that described in more detail elsewhere.^53^ In brief, 38 reflective markers and 4 marker clusters according to the Cleveland Clinic Markers set were used for the kinematic data collection. Kinetic data were recorded for the right and left limbs using two 3D force plates at 2000 Hz embedded in the ground. In Vicon Nexus 2.18, the 3D marker trajectories and kinetic data were then labeled, gap filled, and filtered using a fourth-order low-pass Butterworth filter with a cutoff frequency of 20 Hz for kinematic data and 25 Hz for kinetic data. The vertical force components of the right and left limbs were combined to identify the first ground contact phase after the drop via a threshold of 20 N for initial contact and takeoff ^37^ and to identify the time point of the maximal vertical ground-reaction force.

To assess muscle activity, surface EMG sensors measuring at 2000 Hz (Myon AG) were used. Sensors were placed on the vastus lateralis (at two-thirds on the line from the anterior spina iliaca superior to the lateral side of the patella), vastus medialis (at 80% on the line between the anterior spina iliaca superior and the joint space in front of the anterior border of the medial ligament), biceps femoris (at 50% on the line between the ischial tuberosity and the lateral epicondyle of the tibia), and semitendinosus (at 50% on the line between the ischial tuberosity and the medial epicondyle of the tibia) muscles, as recommended by the SENIAM (surface EMG for noninvasive assessment of muscles) group and as further described at http://www.seniam.org/. The EMG signals were full-wave rectified and filtered with a fourth-order zero-lag Butterworth filter and a bandpass filter, with 20 Hz and 500 Hz taken as cutoff frequencies. As a next step, the filtered EMG signal amplitudes were smoothed using a point-by-point moving symmetric root mean square filter with a time period of 30 ms.^17^ The intensity of the signal was set in relation to the signal of a maximal voluntary contraction (MVC) of the corresponding muscle performed before motion analysis tasks. The MVC was determined during maximal isometric contraction while pushing against a stable resistance for both muscle groups. The MVC of the quadriceps was determined in the seated position with 90° knee flexion, whereas the MVC of the hamstrings was determined in a Nordic hamstring exercise position, which was kneeling at a NordBord Hamstring Testing System (VALD Performance) with a knee angle of 110° (with 180° referring to fully extended knees).

#### Parameter Calculation

The overall quadriceps activity was calculated as the mean muscular activity of the vastus lateralis and vastus medialis muscles; similarly, the overall hamstring activity was calculated as the mean activity of the semitendinosus and the biceps femoris muscles at the time point of maximal ground-reaction force. The quadriceps-to-hamstring activation ratio (QH ratio) was calculated as the MVC-normalized EMG activation of the quadriceps muscles divided by the activation of the hamstrings.^17^ Finally, the lateromedial hamstring activation ratio (LM_ham_ ratio) was calculated as the ratio between lateral and medial hamstring activation at the maximal vertical ground-reaction force. LM_ham_ ratios <1.0 indicated medial hamstring–dominant activation patterns; LM_ham_ ratios >1.0 indicated lateral hamstring–dominant activation patterns.

The medial knee displacement (MKD) before and after fatiguing was determined in accordance with previous definitions.^18^ First, a reference plane connecting the hip, knee joint center, and ankle joint center was defined as 1 frame (0.005 seconds) before the force plate contacted and remained fixed in the hip joint center. The MKD was then calculated as the maximal medial distance (in mm) between the knee joint center and the reference plane throughout the contact phase. A high MKD represents high out-of-plane movement and serves as a proxy for the combination of increased dynamic knee valgus and internal hip rotation, with an ICC of 0.87, indicating good test-retest reliability.^17^

### Statistical Analysis

All the statistical analyses were performed in SPSS (Version 29.0; SPSS Inc). The significance level was set at *P* < .05. The sociodemographic, clinical, radiological, and biomechanical data of the participants were expressed using descriptive statistics. All the data were assessed for normality via the Shapiro-Wilk test. In the case of normality, parametric tests were used. In cases with nonsubstantial deviation from normality,^59^ parametric tests were backed up by bias-corrected accelerated bootstrapping with 10,000 samples.

The main statistical analysis included the following steps. First, to analyze the effectiveness of the fatigue protocol, pre- to postprotocol differences in maximal DJ height were analyzed based on a repeated-measures multivariate analysis of variance (MANOVA) (within-participant factor fatigue [prefatigued, fatigued] and between-participant factor sex [female, male]). Additionally, the mean lactate concentration after the fatigue protocol was reported. Second, to assess sex differences in the bony anatomy of the knee joint (ie, LPTS, MPTS) and the thigh muscle morphology (ie, CSA_ham_, CSA_quad_), a MANOVA with the factor “sex” was performed. To verify that leg dominance did not influence the results, the factor of leg dominance (dominant, nondominant) was used as a covariate in the analysis. For univariate comparisons, Bonferroni correction was applied. Third, to determine the effects of sex and fatiguing state on the EMG activation patterns (ie, quadriceps activity, hamstring activity, QH ratio, LM_ham_ ratio) and the amount of knee valgus (ie, MKD) during DJ landing, a MANOVA with repeated measures was performed (within-participant factor fatigue [prefatigued, fatigued] and between-participant factor sex [female, male]). Again, the influence of the covariate “leg dominance” was assessed and, for univariate comparisons, Bonferroni correction was applied. Fourth, to assess the relationships between the bony anatomy of the knee joint, thigh muscle morphology, thigh muscle EMG activation patterns, and the amount of knee valgus for each potential relationship, Pearson correlation coefficients were calculated for women and men separately. A correlation coefficient with *r* > 0.50 was interpreted as a strong correlation, with *r* values between ±0.30 and ±0.49 considered to indicate a moderate correlation, as suggested by Cohen.^12^

## Results

### Effectiveness of the Fatigue Protocol

DJ height was significantly affected by fatigue (*P* < .001), sex (*P* < .001), and the interaction effect of fatigue and sex (*P* = .03). The participants’ jump heights decreased from 27.6 ± 2.8 cm prefatigued to 25.9 ± 3.5 cm fatigued in female players (*P* = .01) and from 35.9 ± 3.3 cm prefatigued to 32.4 ± 3.0 cm fatigued in male players (*P* < .001). After the fatigue protocol, blood lactate levels reached 8.7 ± 2.2 mmol/l in female players and 10.7 ± 3.3 mmol/l in male players.

### Sex Differences in the Bony Anatomy of the Knee Joint and Thigh Muscle Morphology

At the univariate level, female soccer players had significantly lower CSA_ham_ (*P* < .001) and CSA_quad_ values (*P* < .001) (Table 2). Multivariate analysis revealed a significant effect of sex (*P* < .001), whereas the covariate leg dominance did not significantly affect the results.

**Table 2:** Parameters Related to Bony Anatomy of the Knee Joint and Thigh Muscle Morphology*^*a*^*

	Female	Male	*P* (Sex)
MPTS, deg	5.8 ± 1.8	5.4 ± 1.9	ns
LPTS, deg	6.7 ± 2.5	5.9 ± 2.2	ns
CSA_ham_, mm^2^	28.4 ± 4.7	35.5 ± 5.5	<.001
CSA_quad_, mm^2^	63.5 ± 6.7	80.7 ± 8.5	<.001

a Data are presented as mean ± SD. CSA_ham_, cross-sectional area of hamstring; CSA_quad_, cross-sectional area of quadriceps; LPTS, lateral posterior tibial slope; MPTS, medial posterior tibial slope; ns, not significant.

### Sex Differences and Fatigue-Induced Differences in Thigh Muscle EMG Activation Patterns and Knee Valgus

At the univariate level (Table 3), female soccer players demonstrated significantly greater hamstring activity (*P* = .04), while quadriceps activity was comparable with that of their male counterparts. Consequently, the QH ratio tended to be lower in female than in male players, even though the *P* value of .05 did not reach significance. This means that female soccer players may tend to have less pronounced quadriceps-dominant activation patterns than male soccer players do. Moreover, there were significant sex differences in the LM_ham_ ratio (*P* = .03). Accordingly, female soccer players exhibited medial hamstring–dominant activation patterns (LM_ham_ ratio <1.0), whereas male players tended to exhibit lateral hamstring–dominant activation patterns (LM_ham_ ratio >1.0). With respect to knee kinematics, compared with male soccer players, female soccer players had increased MKD values, indicating increased dynamic valgus (*P* = .02).

**Table 3:** Thigh Muscle EMG Activation Patterns and MKD During Drop Jumps in the Prefatigued and Fatigued States*^*a*^*

	Female	Male	*P* (Sex)	*P* (Fatigue)
Quadriceps activity, % MVC				
Prefatigued	64.6 ± 12.2	66.3 ± 6.9	ns	ns
Fatigued	61.7 ± 15.3	59.1 ± 15.8		
Hamstring activity, % MVC				
Prefatigued	36.2 ± 12.5	30.2 ± 10.5	.04	ns
Fatigued	34.3 ± 9.7	27.7 ± 10.6		
LM_ham_, ratio				
Prefatigued	1.0 ± 0.4	1.3 ± 0.9	.03	ns
Fatigued	0.8 ± 0.4	1.3 ± 0.7		
QH, ratio				
Prefatigued	2.0 ± 0.8	2.5 ± 0.9	ns	ns
Fatigued	2.0 ± 1.0	2.5 ± 1.4		
MKD, mm				
Prefatigued	28.9 ± 17.6	20.3 ± 11.6	.02	ns
Fatigued	26.3 ± 14.0	18.0 ± 13.2		

a Data are presented as mean ± SD. EMG, electromyography; LM_ham_, lateromedial hamstring activation; MKD, medial knee displacement; MVC, maximal voluntary contraction; QH, quadriceps-to-hamstring activation; ns, not significant.

With respect to thigh muscle EMG activation patterns (quadriceps activation, hamstring activation, the LM_ham_ ratio, and the QH ratio) and knee valgus (MKD), sex had a significant effect on the multivariate analysis (*P* = .03) but not fatigue. The covariate leg dominance did not significantly affect the results.

### Relationships Between PTS Characteristics, Thigh Muscle Morphology, Thigh Muscle Activation Patterns, and Dynamic Knee Valgus

**Figure 4.**
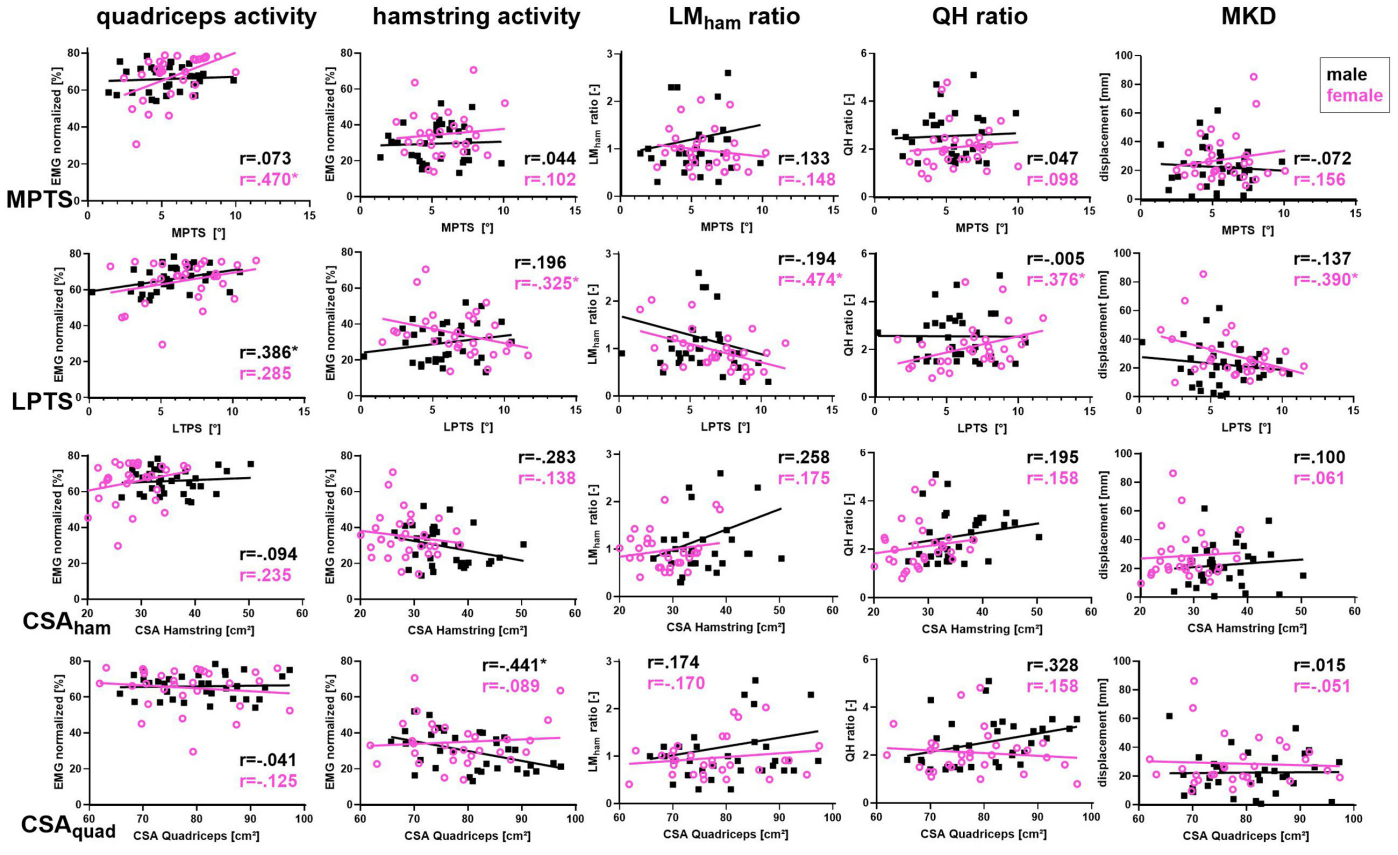
Relationships between anatomic parameters and functional parameters for female (pink circles) and male (black solid squares) soccer players. Asterisks indicate potentially relevant relationships (*P* < .10). CSA_ham_, cross-sectional area of hamstring; CSA_quad_, cross-sectional area of quadriceps; EMG, electromyography; LM_ham_, lateromedial hamstring activation; LPTS, lateral posterior tibial slope; MKD, medial knee displacement; MPTS, medial posterior tibial slope; QH, quadriceps-to-hamstring activation.

Analysis of the relationships for both sexes revealed that female and male soccer players had several associations with the investigated parameters (Figure 4).

Among female soccer players, the MPTS was positively correlated with quadriceps activity (*r* = 0.470; *P* = .009). Moreover, the LPTS of female soccer players was, by trend, negatively correlated with hamstring activity (*r* = −0.325; *P* = .08), and there was a moderate positive correlation between LPTS and QH ratio (*r* = 0.376; *P* = .04). Additionally, there was a negative correlation between LPTS and the LM_ham_ ratio (*r* = −0.474; *P* = .008) and between LPTS and MKD (*r* = –.390; *P* = .03).

In male soccer players, the analysis revealed only a moderate positive correlation between LPTS and quadriceps activity (*r* = 0.386; *P* = .03) and an inverse correlation between CSA_quad_ and hamstring activity (*r* = 0.441; *P* = .01).

## Discussion

The key findings of this study were as follows: (1) There was a significant sex difference in thigh muscle morphology, with female players having significantly smaller thigh muscles, but there was no sex difference in PTS characteristics; (2) during DJ landing, female soccer players demonstrated significantly greater hamstring activity, while quadriceps activity was comparable with that of their male counterparts. There were significant sex differences in the LM_ham_ activation ratio, with female players exhibiting greater medial than lateral hamstring activation (ratio <1.0), whereas male players presented opposite activation patterns (ratio >1.0). MKD was greater in female players than in male players. No fatigue-induced differences were observed. (3) In female soccer players, the steeper their LPTS was, (A) the larger their QH ratio and the smaller their hamstring activity, (B) the smaller their LM_ham_ ratio, and (C) the lower their dynamic valgus. In male players, a steeper LPTS was associated with greater quadriceps activity only.

### Sex Differences in PTS Characteristics, Thigh Muscle Morphology, Thigh Muscle Activation Patterns, and Dynamic Knee Valgus

From an anatomic/morphological perspective, there are 2 key findings of this study. First, similar to the results of a large-scale cadaveric study, the MPTS and LPTS did not significantly differ between the sexes in our study.^11^ Second, female soccer players were found to have significantly smaller CSA_ham_ and CSA_quad_ values than male soccer players did, suggesting sex differences in thigh muscle size. This is certainly not a novel observation, as it is well known that males have greater overall and regional skeletal mass than females do,^1,21,39^ which holds true for both absolute and relative muscle mass.^21,39^

In addition to muscle morphology, neuronal muscle activation is a main factor for muscle force generation.^30^ During DJ landing, the female soccer players in our study demonstrated significantly greater hamstring activity, whereas quadriceps activity was comparable with that of their male counterparts. Thus, female soccer players may tend to have less pronounced quadriceps-dominant activation patterns than male soccer players do. This finding is in line with a previous study in youth competitive alpine skiers, which reported a significantly lower quadriceps-to-hamstring activation ratio during DJ landing in girls than boys.^17^ However, our findings contradict observations in cutting maneuvers (ie, unilateral landings after stepping sideward), in which female players demonstrated less hamstring activity.^16^

In terms of muscle strength, which was not assessed in this study but is mainly determined by the measured muscle morphology and neuronal muscle activation patterns, it is well-documented that males have higher absolute and relative strength values than females do.^21,43,50^ This also applies to the relative maximal eccentric hamstring strength,^43^ which is crucial for counteracting the anterior shear force on the tibia that results from aggressive quadriceps loading during mechanisms leading to ACL injuries.^14^ In this context, the increased activation of the hamstrings during DJ landing in female athletes could indicate a coping strategy of the neuromuscular system to compensate for their lower relative hamstring muscle mass while still generating the muscle forces required for adequate knee stabilization; however, this is just a hypothesis at this stage and further research on this topic is needed.

Two further interesting findings of our study were (1) that female soccer players typically exhibited medial hamstring–dominant activation patterns, whereas male players presented lateral hamstring–dominant activation patterns, and (2) that the dynamic valgus motion during DJ landing was greater in female soccer players than in male soccer players, which further supports the findings of previous studies.^13,18,22,31,48^ Similarly, in a recent study with competitive alpine skiers, we reported that the semimembranosus muscle, as a medial hamstring muscle, accounts for a greater proportion of the overall hamstring size in women than in men.^21^ Thus, given that the medial hamstrings serve the function of internal rotation of the knee, which, together with dynamic knee valgus, is one of the key components of ACL injury mechanisms, this may have implications for establishing sex-specific injury prevention programs. However, further research on this topic is warranted.

### No Fatigue-Induced Differences in Thigh Muscle Activation Patterns or Dynamic Knee Valgus

Fatigue had no effect on relative quadriceps or hamstring activation during DJs, even though blood lactate was significantly increased and jump height significantly decreased after the fatigue protocol. These results are in line with those of a previous study,^47^ which also showed similar EMG amplitudes with fatigue, suggesting that neuromuscular modular organization is the building block of human movement. Furthermore, in our study with adult elite soccer players, fatigue had no influence on the magnitude of dynamic knee valgus, whereas a study using a similar fatigue protocol in adolescent athletes reported increased valgus in the fatigued state.^20^ The exact reason for this difference remains unclear; however, it could be speculated that the same fatigue protocol had different effects on adolescent athletes than on better-trained elite athletes.

### PTS Characteristics and Their Relationships With Thigh Muscle Morphology, Activation Patterns, and Dynamic Knee Valgus

A steeper tibial slope has been reported to be a significant anatomic risk factor for noncontact ACL injuries,^15,23,26,61^ as both anterior tibial translation and internal rotation of the tibial plateau are influenced by the extent of the PTS.^5,32,55^ Our study revealed that, in female soccer players, the steeper their LPTS was, (1) the larger their QH ratio and the smaller their hamstring activity were, (2) the smaller their LM_ham_ ratio was, and (3) the lower their dynamic valgus was. In male players, a steeper LPTS was associated with greater quadriceps activity only.

Accordingly, these findings, together with our additional findings of a positive correlation of MPTS and LPTS with quadriceps activity in female and male players, respectively, further support the anterior tibial translation–increasing effect of steeper PTS shown in cadaveric knees,^5^ as increased proportional quadriceps activity is known to be related to increased anterior tibial translation.^58^ Moreover, our findings of negative correlations between LPTS and the LM_ham_ ratio and between LPTS and MKD suggest that increased PTS on the lateral side reduces the occurrence of dynamic knee valgus, making a coping strategy to unload/protect the ACL (ie, a more pronounced lateral hamstring muscle co-activation, corresponding to a larger LM_ham_ ratio) unnecessary.^24^

### Clinical Implications

The findings of the current study further highlight the importance of considering the bony anatomy of the knee joint in the design of neuromuscular prevention and rehabilitation, or the choice of graft type. In particular, soccer players with steeper PTSs may benefit from preventative training of the hamstring muscles; however, in view of the typical loading patterns during noncontact ACL injury mechanisms,^9,14,33,46^ the loading pattern should progress from basic hamstring strength exercises to eccentric, maximal, and rapid activation. Moreover, female soccer players may benefit from leg axis stability training focusing on eccentric, maximal, and rapid activation of the lateral hamstrings (and hip external rotators^38^), because they may be more prone to dynamic knee valgus. The findings of the present study may also have potential implications for the choice of graft type for primary and secondary ACL reconstruction using autologous tendons considering the evaluation of an individualized approach.^4^ An ACL graft from the extensor group, such as the quadriceps and patellar tendons, provides excellent results in ACL reconstruction,^41,42^ reducing the potential impairment of active translational (and rotational) stabilization by sparing the medial hamstring tendons.^57^ Moreover, given the high risk of primary and secondary ACL injury in pivoting-sports athletes and female soccer players in particular,^3,7,19^ the results of the current study may provide further understanding of the importance of the hamstring muscles, particularly in the female population, especially in those with unfavorable knee anatomy. However, despite these novel functional insights regarding the bony anatomy of the knee joint and its relationship with measures related to knee stabilization in healthy soccer players, future studies are needed to assess the effect of sex- and/or knee anatomy–dependent graft choice on clinical outcomes in patients undergoing ACL reconstruction and to provide more evidence-based recommendations.

### Limitations and Methodological Considerations

This study has several limitations that should be considered when interpreting the study findings. First, the manual, radiologically based determination of anatomic parameters such as PTS may be examiner dependent. To counteract this, these measures were determined by 3 independent raters, and the corresponding mean values were used for further analysis. Second, the fatigue protocol was an attempt to create preloading comparable with fatigue in a soccer match. Although a substantial increase in blood lactate and a decrease in jump height indicate fatigue, the fatigue protocol used could have been more soccer specific. Moreover, the soccer players in this study may have partially recovered from the fatigue protocol during the postfatigue training trials; however, the decrease in jump height at retest suggested a significant fatigue effect that lasted throughout the test-retest period. Third, the correlations between anatomic and functional parameters observed in this study were (despite being significant) *moderate* only. Accordingly, further studies with larger samples and/or other study populations may be needed to confirm their generalizability.

## Conclusion

In soccer players, PTS characteristics and thigh muscle morphology, as well as thigh muscle activation patterns and dynamic knee valgus during DJ landing, are sex dependent, and there are distinct associations between PT characteristics and measures related to neuromuscular control of the knee joint. Thus, the findings of the current study may further highlight the importance of also considering sex and the bony anatomy of the knee joint in the design of neuromuscular prevention and rehabilitation or the choice of graft type. However, future studies are needed to assess the effects of sex- and/or knee anatomy–dependent prevention efforts or graft choice on clinical outcomes.
